# Attention deficit hyperactivity disorder in adulthood and the heritability of this condition

**DOI:** 10.1192/j.eurpsy.2021.1638

**Published:** 2021-08-13

**Authors:** J. Borges, R. Almeida Leite, M. Almeida, S. Morais, N. Madeira

**Affiliations:** 1 Departamento De Psiquiatria E Saúde Mental, Centro Hospitalar do Baixo Vouga, Aveiro, Portugal; 2 Centro De Responsabilidade Integrado De Psiquiatria, Centro Hospitalar e Universitário de Coimbra, Coimbra, Portugal

**Keywords:** heritability, attention deficit hyperactivity disorder, Adult

## Abstract

**Introduction:**

Attention Deficit Hyperactivity Disorder (ADHD) is a common neurodevelopmental disorder characterized by inattention and/or hyperactivity-impulsivity resulting from the interaction of genetic and environmental risk factors. Family studies shows that persistent ADHD is very familial.

**Objectives:**

We aim to review the literature on this condition and its heritability and describe the implications that a possible misdiagnosis can have during life.

**Methods:**

Bibliography review was performed using the databases PubMed and Cochrane, using the following keywords: “ADHD”; “Adults”; “Heretability”; “Family” and “Rater effect”.

**Results:**

Childhood ADHD persists into adolescence and adulthood substantially, identified in some studies, as going up to 78%. The prevalence of ADHD in children and adults is between 2.5% and 5% worldwide. Family studies have shown that children of adults with ADHD are at higher risk of having ADHD. Some large-scale twin studies of adult ADHD, used self-report assessments of ADHD symptoms and estimated the heritability of this condition to be between 30 to 40%, which differs from other studies that analyse parents and teachers responses and estimates heritability to be between 60 and 90%.

**Conclusions:**

Since there is a direct influence of the evaluators in estimating the extent of ADHD heritability, future studies need to clarify and describe in detail all the related characteristics of the raters. Although ADHD is widely studied, there is still a lot to learn about its etiology. The diagnosis of ADHD is clinical and complex and must be considered both in childhood and adolescence and in adulthood, with special emphasis on the family antecedents.

**Disclosure:**

No significant relationships.

